# Large Region of Homozygous (ROH) Identified in Indian Patients with Autosomal Recessive Limb-Girdle Muscular Dystrophy with p.Thr182Pro Variant in *SGCB* Gene

**DOI:** 10.1155/2023/4362273

**Published:** 2023-03-28

**Authors:** V. Manjunath, S. G. Thenral, B. R. Lakshmi, Atchayaram Nalini, A. Bassi, K. Priya Karthikeyan, K. Piyusha, R. Menon, A. Malhotra, L. S. Praveena, R. M. Anjanappa, S. M. Sakthivel Murugan, Kiran Polavarapu, Mainak Bardhan, V. Preethish-Kumar, Seena Vengalil, Saraswati Nashi, S. Sanga, M. Acharya, R. Raju, V. R. Pai, V. L. Ramprasad, R. Gupta

**Affiliations:** ^1^MedGenome Labs Pvt. Ltd., Bangalore, India; ^2^Yenepoya Medical College, Yenepoya (Deemed to be University), Deralakatte, Mangalore, India; ^3^Molecular Diagnostics Counseling Care and Research Centre (MDCRC), Coimbatore, Tamil Nadu, India; ^4^National Institute of Mental Health and Neurosciences, Bangalore, India; ^5^National Institute of Biomedical Genomics, Kolkata, India

## Abstract

The sarcoglycanopathies are autosomal recessive limb-girdle muscular dystrophies (LGMDs) caused by the mutations in genes encoding the *α*, *β*, *γ*, and *δ* proteins which stabilizes the sarcolemma of muscle cells. The clinical phenotype is characterized by progressive proximal muscle weakness with childhood onset. Muscle biopsy findings are diagnostic in confirming dystrophic changes and deficiency of one or more sarcoglycan proteins. In this study, we summarized 1,046 LGMD patients for which a precise diagnosis was identified using targeted sequencing. The most frequent phenotypes identified in the patients are LGMDR1 (19.7%), LGMDR4 (19.0%), LGMDR2 (17.5%), and MMD1 (14.5%). Among the reported genes, each of *CAPN3*, *SGCB*, and *DYSF* variants was reported in more than 10% of our study cohort. The most common variant *SGCB* p.Thr182Pro was identified in 146 (12.5%) of the LGMD patients, and in 97.9% of these patients, the variant was found to be homozygous. To understand the genetic structure of the patients carrying *SGCB* p.Thr182Pro, we genotyped 68 LGMD patients using a whole genome microarray. Analysis of the array data identified a large ~1 Mb region of homozygosity (ROH) (chr4:51817441-528499552) suggestive of a shared genomic region overlapping the recurrent missense variant and shared across all 68 patients. Haplotype analysis identified 133 marker haplotypes that were present in ~85.3% of the probands as a double allele and absent in all random controls. We also identified 5 markers (rs1910739, rs6852236, rs13122418, rs13353646, and rs6554360) which were present in a significantly higher proportion in the patients compared to random control set (*n* = 128) and the population database. Of note, admixture analysis was suggestive of greater proportion of West Eurasian/European ancestry as compared to random controls. Haplotype analysis and frequency in the population database indicate a probable event of founder effect. Further systematic study is needed to identify the communities and regions where the *SGCB* p.Thr182Pro variant is observed in higher proportions. After identifying these communities and//or region, a screening program is needed to identify carriers and provide them counselling.

## 1. Introduction

Limb-girdle muscular dystrophies (LGMDs) are a heterogeneous group of disorders leading to progressive muscle wasting and weakness, predominantly characterized by limb-girdle weakness. It is caused by mutations in 32 genes causing LGMD. Sarcoglycanopathies (SG) are the most frequent form of autosomal recessive LGMD comprising of four subtypes, LGMDR3, LGMDR4, LGMDR5, and LGMDR6, caused by mutations in *SGCA*, *SGCB*, *SGCG*, and *SGCD* encoding for the alpha-, beta-, delta-, and gamma-sarcoglycan proteins, respectively [1]. These transmembrane glycoproteins provide stability by forming a tetrameric complex to dystrophin-dystroglycan complex (DGC), which acts as a linker between the extracellular matrix and the muscle cytoskeleton [2–4]. Mutations in any one of the genes can lead to the loss of membrane integrity leading to the clinical phenotype. The diagnosis is based on immunohistochemistry of the muscle biopsy, and in recent years, genetic testing is due to advance high-throughput next-generation genome sequencing [5, 6].

The subtypes of sarcoglycanopathy vary in prevalence according to ethnicity and geographic region. The overall prevalence is estimated for LGMDR3, LGMDR4, and LGMDR5 as 0.9, 0.016, and 0.22 per million, respectively [1, 7–9]. The frequency of LGMDR6 appears to be very rare worldwide. Severe childhood-onset LGMD is primarily associated with mutations of *SGCG*, *SGCA*, *SGCB*, or *SGCD* genes. Available studies on sarcoglycanopathies from India have been largely based on immunocytochemical characterization. Even though in recent years genetic analysis of LGMDs has increasingly been undertaken in various parts of India, there are only a few genetically confirmed SG patients with one small series and some case reports available [10–13].

The clinical-genetic overlap among subtypes and with other neuromuscular diseases complicates disease-subtype identification, lengthening the diagnostic process. These efforts are very limited in understanding the prevalence pattern of these diseases in our large country with a diverse population. Identifying any founder events within subpopulation of these diseases will be very beneficial in genetically informed risk stratification and management. Founder mutations are believed to contribute more to the burden of recessive diseases than consanguinity alone in certain Indian populations. The long-term continuation of the traditional practice of intracaste endogamy has contributed significantly to the excessive cases of recessive disease in India [14, 15]. In this study, we first describe the genes identified in 1,168 LGMD patients and then performed the phenotype and genotype correlations. Furthermore, we perform an additional analysis of patients carrying *SGCB* p.Thr182Pro, which is the more frequent pathogenic variant identified in our study cohort. The *SGCB* variant was also reported to be the most frequent in our recent study with different sarcoglycanopathies [13]. Analysis identified a large ROH region shared across the patients with *SGCB* p.Thr182Pro variant.

## 2. Materials and Methods

### 2.1. Retrospective Data of LGMD Patients, Samples, and Ethical Compliance

We analyzed the prioritized gene variants reported in our lab for 1,046 LGMD patients received over a period of the last ~6 years (June 2015–March 2021). The patient samples from MDCRC (Molecular Diagnostics, Counselling, Care and Research Center) at Coimbatore, Tamil Nadu, India, contributed ~24.8% of the total diagnosed LGMD cases. This was followed by a multidisciplinary neuromuscular disease clinic of a quaternary center of national importance, which contributed ~20.2% of the total LGMD patients. This clinic is part of the national referral center for neurological disorders in Bangalore, India. The remaining 55.9% of the patient samples came from different clinics spread all over India. To understand the genetic structure of the patient that carries the most recurrent variant (*SGCB* p.Thr182Pro; chr4:g.52028807T>G) in our cohort, we genotyped 68 patients using a whole genome array.

### 2.2. Sample Processing

DNA was extracted from the whole peripheral blood using QIAsymphony (QIAGEN Inc.) extraction automation system. Further, DNA concentrations were measured by a fluorometer. Purified 100 ng of genomic DNA was subjected to mechanical fragmentation by Covaris to obtain an average size of 200 bp of DNA fragments. The fragmented DNA of each sample was put through end repair, adenylation, adaptor ligation, and amplification to obtain whole genome libraries using the Kapa HTP library preparation kit (KAPA Biosystems, USA). These libraries were hybridized with biotin-labelled custom-designed exome capture probes (NimbleGen, Roche); after 16 hours of temperature depending on hybridization, whole exome targets were captured using streptavidin beads by temperature wash (NimbleGen, Roche). The libraries were then sequenced to mean > 80–100x coverage on 150^∗^2 Illumina sequencing platform (Hiseq2500 and HiSeqX, Illumina Inc.).

### 2.3. NGS Data Processing

Following quality check and adapter trimming using fastq-mcf (version 1.04.676), the sequencing reads obtained are aligned to the human reference genome (GRCh37/hg19). The aligned reads were sorted, duplicate reads were removed, and the variants were called using the GATK best practices pipeline using Sentieon (v201808.07). Gene annotation of the variants was performed using the VeP program against the Ensembl release 91 human gene model. The variants were annotated for allele frequency (population databases—gnomAD (v3.0), ExAC, 1000Genome, and MedGenome population-specific database), in silico prediction tools (CADD, PolyPhen-2, SIFT, Mutation Taster2, and LRT), and disease databases (OMIM, ClinVar, and HGMD). The clinically significant variants were sequentially prioritized and analyzed using Varminer (MedGenome variant interpretation tool). In addition to single nucleotide variants (SNVs) and small indels, copy number variants (CNVs) are detected from targeted sequence data using the ExomeDepth (v1.1.10) method. The variants in genes correlating the disease phenotype and inheritance were prioritized. Clinical interpretation of the variants was assigned based on ACMG guidelines [16].

### 2.4. Sanger Sequencing

The variant was confirmed by PCR amplification of exon 4 of the SCGB gene by gene-specific primers (PXL-A0200614, Pxlence) in 7 samples followed by Sanger sequencing ABI 3730 genetic analyzer (Applied Biosystems, CA).

### 2.5. Array Data Processing

Following the manufacturer's protocol, samples were analyzed using the Illumina Infinium Global Screening Array-24 v3.0 Kit and genome build GRCh37/hg19. All data collected were evaluated using Illumina's GenomeStudio v2.0 software. Genotypes obtained from the Illumina Global Screening Array were used to identify haplotype groups. We generated the Global Screening Array version 3 (Illumina Inc.) data of 206 samples. PLINK v1.90 was used to retain the biallelic SNPs and nonindels [17]. We applied a filter of 10-6 for the Hardy-Weinberg equilibrium and retained all samples having greater than 95% genotyping rate. We performed the pihat analysis using the genome option of PLINK v1.90. Additionally, duplicated samples were removed for runs of homozygosity analysis and haplotype analysis. For IBD and ancestry analysis, the duplicates and first-degree related samples were removed.

#### 2.5.1. Haplogroup Analysis

We have used the joint vcf from the genotype data generated using Global Screening Array version 3 (Illumina Inc.) of 187 samples to predict the maternal haplogroup using the haplogrep v2.4.0 tool [18], and paternal haplogroups are predicted using the inhouse script which queries the ISOGG (http://www.isogg.org/tree/) reference file [19].

#### 2.5.2. Admixture Analysis

We used the admixture tool, to estimate the ancestry in global ancestry components for the Global Screening Array version 3 samples. For admixture analysis, we inferred the population substructure using a reference dataset from GenomeAsia pilot project data, from which we included representatives from West Eurasia, South Asia, Africa, Southeast Asia, Northeast Asia, and Oceania.

#### 2.5.3. Principal Component Analysis

Principal component analysis was used to stratify a cohort of 187 samples using a reference dataset from GenomeAsia pilot project data. SNPRelate package in R version 3.3.1 was used to do the principal component analysis [20].

#### 2.5.4. ROH Analysis

The ROH analysis was performed on the QC-passed samples (autosomes only) in PLINK v1.90 with parameters homozyg, homozyg-window-snp 50, homozyg-snp 50, homozyg-window-missing 3, homozyg-kb 100, and homozyg-density 1000. The ROH pairs were summarized separately for cases and controls.

#### 2.5.5. PHASE Haplotype Analysis

The haplotype analysis was performed using PHASE v2.1.1 [21]. The region of interest was extracted from QC-passed VCF. With the case-control status incorporated in the PED and MAP file, IPGWAS tool was used for conversion to the input file of PHASE [22]. PHASE was run by taking into consideration the case-control status of the samples and a summary of haplotypes was prepared.

## 3. Results

### 3.1. Retrospective Analysis of LGMD Patients

**Figure 1:**
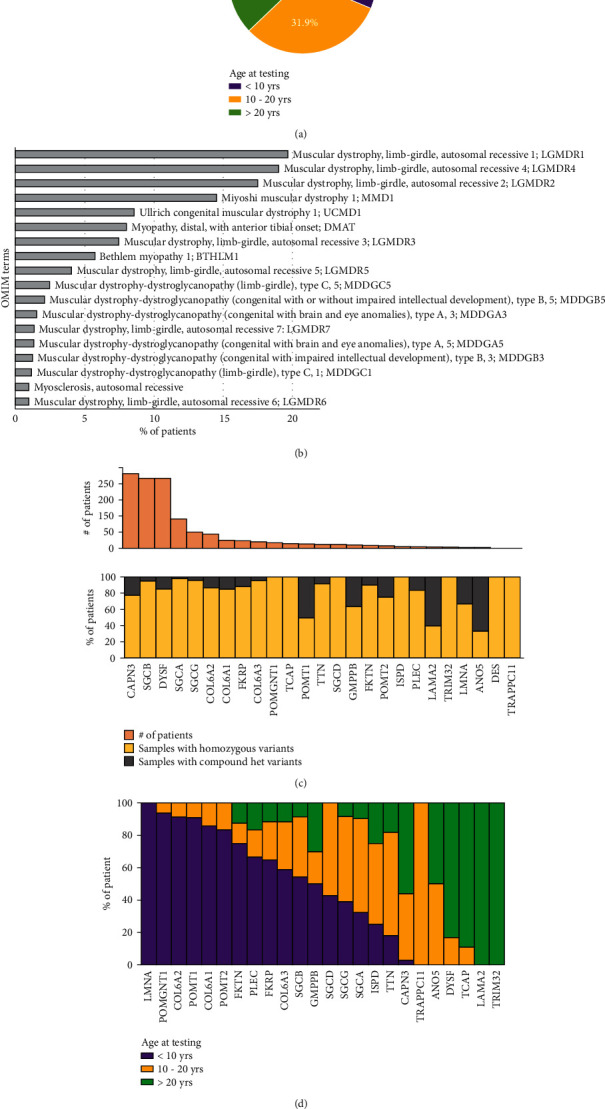
LGMD study cohort. (a) Distribution of patient age at genetic testing (<10 yrs, 10-20 yrs, and >20 yrs). (b) Most common OMIM term assigned to the patient in our study cohort. LGMDR1, LGMDR4, LGMDR2, and MMD1 are the most common symptoms/phenotype reported in the patient. (c) Frequency of pathogenic variant identified in 25 genes. (d) Gene reported and age of patient at genetic testing. Pathogenic variants in *POMGNT1*, *COL6A1/2*, and *POMT1* genes are found in younger affected individuals (<10 yrs) whereas pathogenic variants in *DYSF*, *CAPN3, LAMA2*, and *TTN* genes are found in individuals with age > 10 yrs.

We analyzed the significant (pathogenic/likely pathogenic) variants reported in 1,046 LGMD patients sequenced using targeted panels and exomes over a period of ~6 years (June 2015–March 2021). All these patients carry a pathogenic/likely pathogenic variant among the 42 genes recommended by the 229th ENMC international workshop on Limb-girdle muscular dystrophies - Nomenclature and reformed classification Volker Straub and Cohen et al. [23, 24]. For 85.7% (*n* = 907) of the patients, sex information was available. Among these, 65.2% (*n* = 585) and 34.8% (*n* = 312) patients are male and female, respectively. The age at which the clinical testing was performed was available for ~72% (*n* = 753) of the patients. Among these, 31.2% of patients have an age < 10 yr and 31.9% age 10–20 yr, and remaining 36.9% of the patients have age > 20 yrs at the time of testing (Figure 1(a)). The phenotype/symptoms of the patients were captured using OMIM terms. Overall, 50 different OMIM terms were mapped to the study cohort patients (Supp. Table S1). More than one phenotype term was assigned in ~27% of the patients. The most common OMIM phenotype terms assigned in our study cohort included LGMDR1 (19.7%), LGMDR4 (19.0%), LGMDR2 (17.5%), and MMD1 (14.5%) (Figure 1(b)).

In our study cohort, we found pathogenic variants in 25 different genes (Figure 1(c)). Among all patients, in 915 (87.5%), we found a pathogenic homozygous variant, and in the remaining 131 (12.5%) patients, we found compound heterozygous variants. Among these, *CAPN3* (22.0%), *SGCB* (20.7%), *DYSF* (20.6%), *SGCA* (8.7%), and SGCG (4.8%) are the top 5 frequently reported genes in our LGMD cohort (Supp. Table S2). We further looked at the genes prioritized and the age at which the genetic diagnosis was performed. Pathogenic mutations in *POMGNT1*, *COL6A2*, *POMT1*, *COL6A1*, *FKRP*, *COL6A3*, *SGCB*, and *GMPPB* genes were more commonly found in patients at younger age < 10 years whereas *DYSF*, *CAPN3, LAMA2, TTN,* and *SGCA* genes were more commonly found in the patients with age > 10 years (Figure 1(d)). Pathogenic variant in *SGCG* gene was found to be in equal proportion for patients above and below the age of 10 years at the time of genetic testing.

Among the reported pathogenic variants, *SGCB* p.Thr182Pro is the most frequently reported variant in our study cohort. This variant was found in 146 (12.5%) patients, and in 142 (97.9%) of them, it was found to be homozygous. We recently published this variant in our study on 20 patients, and it was also reported as one of the most frequent variants [13]. To investigate the patients with *SGCB* p.Thr182Pro variant, we performed whole genome array genotyping and analysis to understand the founder event, which is described below.

### 3.2. Ancestry Analysis

**Figure 2:**
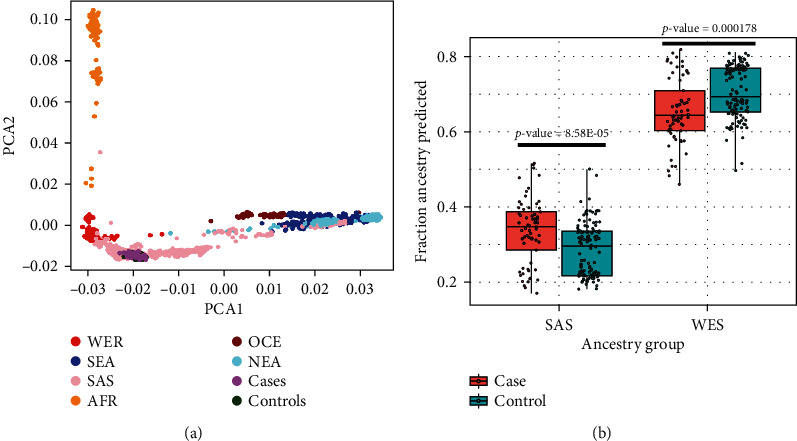
Ancestry analysis. (a) PCA value distribution of our study cases and controls overlaid with Genome Asia study. Our cases and control samples overlap South Asian ancestry data. (b) Proportion of South Asian (SAS) and West Eurasian (WES) predicted for case and control samples using admixture analysis. The difference in SAS and WES admix proportion between case and control groups was found to be significant (*t*-test, SAS = 8.58*E* − 05, WES = 0.000178).

Ancestry analysis of QC-passed 196 samples (case = 68, control = 128) using PCA revealed that all samples from both the control and case group belong to the South Asian (SAS) ancestry when compared with the GenomeAsia (GAsP) study (Figure 2(a)) [25]. We then performed an admixture analysis to estimate different ancestry fractions in each sample [26]. As expected, a higher proportion of ancestry South Asian (SAS) was observed in both the case and control groups [25, 27]. However, we observed that cases have a lower SAS and higher WER (West Eurasian/European) proportion as compared to the controls (unpaired *t*-test, *p* value < 0.0001) (Figure 2(b)). The median ancestry proportion of SAS observed in case and control was 0.644, and 0.694, respectively. In contrast, a median WER proportion of 0.347 and 0.296 was observed in the case and control, respectively. The difference in SAS and WES admixture proportion between case and control was found to be statistically significant (*t*-test, SAS *p* value = 8.58*E* − 05, WES *p* value = 0.000178). We further analyzed the maternal and paternal haplogroups of the case and control groups (Supp. Table S3). M maternal haplogroup was found to be the most frequent (12.12%) in cases compared to the controls (7.37%) (chi-square, *p* -value = 0.279). In the control group, M5 maternal haplogroup was found to be the most frequent (13.11%) compared to the case group with 7.57% (chi-square, *p* value = 0.250) (Supp. Table S3a). Paternal haplogroup analysis showed that H1a1 is most frequent in both the case (28%) and control (20.4%) groups whereas R1a1 haplogroup is more frequent among cases (20%) compared to 8.16% seen in the control group (chi-square, *p* -value = 0.091) (Supp. Table S3b).

### 3.3. Runs of Homozygosity (ROH) Analysis

**Figure 3:**
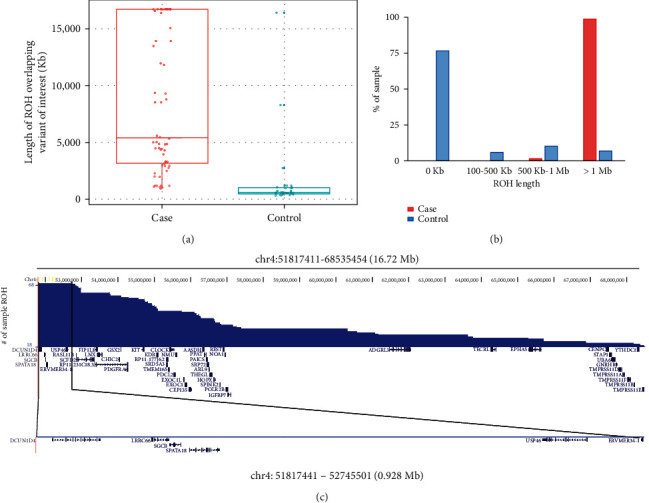
ROH analysis. (a) Distribution of ROH (region of homozygosity) length (in kb) identified in case and control samples overlapping variant of interest (*SGCB* p.Thr182Pro). (b) Majority of cases contain a large ROH region (~1 Mb) overlapping variant of interest. (c) Length and frequency of ROH identified in the cases. The longest ROH extends up to 16.72 Mb which covers 2,929 markers and encompasses 50 protein-coding genes.

Using plink, we performed ROH analysis of 196 samples (case = 68, control = 128). ROH in samples and overlapping variant (chr4:g.52028807T>G) sites was taken up for further analysis. We observed large ROH regions in cases compared to the controls (Figure 3(a)). In the majority (75%) of the control samples, we did not find any ROH overlapping with the variant of interest. In only 7% (*n* = 7) of the controls, we found ROH with ≥1 Mb (Supp. Table S4), whereas in almost all cases (67 out of 68), we found ROH ≥ 1 Mb (Figure 3(b)). The common ROH region identified across all cases is 928,060 bp (chr4:51817441-52745501, GRCh38 coordinate) and consists of 119 markers from the array (Figure 3(c)). The common ROH region encompasses 6 protein-coding genes—*DCUN1D4*, *LRRC66*, *SGCB*, *SPATA18*, *USP46*, and *ERVMER34-1*. We found 4 ROH regions repeated in more than one sample. The most frequent ROH was seen in 18 different samples and is also the longest ROH identified in cases (16.72 Mb) (Figure 3(c)). It consists of 2,929 markers and encompasses 50 protein-coding genes.

### 3.4. Haplotype Analysis

**Figure 4:**
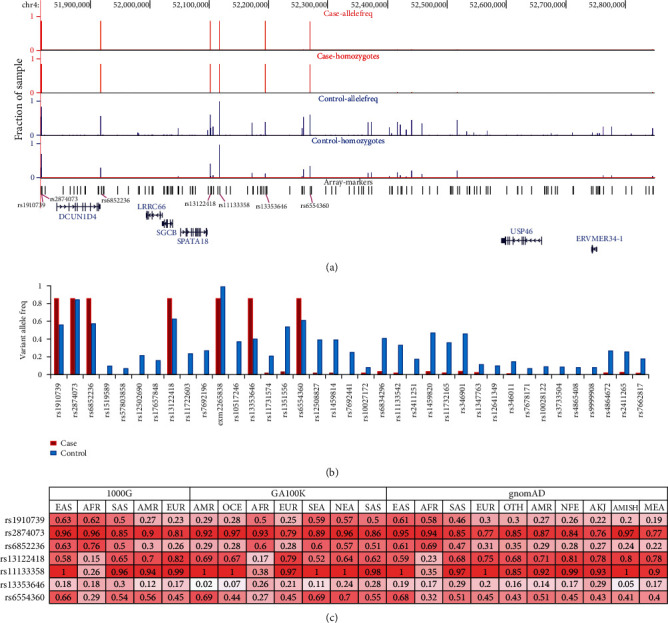
Haplotype analysis. (a) Frequency of 133 array markers and zygosity in case and control identified using haplotype analysis. Cases are shown in red and control is shown in blue. (b) Frequency of 37 markers with allele fraction ≥ 5% in either case or control group. (c) Markers (*N* = 7) with major alleles in the case group and its frequency in different subpopulation groups of 1000G, GA100K, and gnomAD.

Haplotype analysis of the markers around the common ROH region (chr4:51817441-52745501, GRCh38 coordinates) was performed in case (*N* = 68) and control (*N* = 128). Our analysis revealed 133 marker haplotypes (chr4:51817441-528499552) present as a double allele in 58 out of 68 cases (85.3%), and in 2 cases, it was present as a single allele (Figure 4 and Supp. Table S5). The haplotype was present only as a single allele in 32 out of 128 controls. None of the control samples have a double allele. In the 133 markers list, we found 7 markers that had an alternative allele as the major allele in the case group (Figures 4(a) and 4(b) and Supp. Table S6). Of these, we found 5 markers (rs1910739, rs6852236, rs13122418, rs13353646, and rs6554360) of allele frequency to be statistically higher in cases compared to control and overall allele frequency from population databases (Table 1 and Figure 4(b)). We further investigated these 5 markers at the subpopulation level (Figure 4(c)). Interestingly, we found only one marker—rs13353646 (0.28–0.3)—among these to be most common in the South Asian population group. We found that markers rs1910739 (0.57–0.63) and rs6554360 (0.66–0.7) had the highest allele frequency in East, Southeast, and Northeast Asian population groups. Interestingly, rs6852236 (0.6–0.69) was seen most in the African population group, whereas rs13122418 (0.7–0.82) was seen most common in European population group.

## 4. Discussion

Sarcoglycanopathies are caused by mutations which occur in LGMD genes SGCA, SGCB, SGCG, and SGCD that lead to misfolding, truncated, or loss of protein of *α*, *β*, *γ*, and *δ* protein which stabilizes the sarcolemma of muscle cells. The common LGMD symptoms are more similar to DMD, it includes calf hypertrophy, difficulty in climbing, running, scapular wing, and elevated serum creatine kinase levels. Individuals carrying mutations in these SGC genes lead to exhibit symptoms in childhood and vigorous progress in their symptoms by mid teen age. Proximal muscle weakness is predominant in LGMD4 patients [28].

From India, only a few LGMD studies have been reported and thus the available epidemiological data. One of the moderately large cohorts of genetic myopathy cases had a diagnostic yield of 5% for LGMD, of which 2% mutations were in *SGCB* gene [29]. In this study, we summarized the largest study of LGMD (*n* = 1,046) patients from India in which our diagnostic pipeline reported significant (pathogenic/likely pathogenic) variants. The identified pathogenic variants mapped to 25 different genes (Figure 1(c)). The findings from our study replicate what is known about the commonly mutated genes in all LGMD. As reported in the past, we also find that *CAPN3* and *DYSF* are the most frequently mutated gene [30, 31]. *SGCB* is the second highest mutated genes in our cohort and is something which is not reported in the past. The *SGCB* p.Thr182Pro variant is more frequent and is present in 146 (12.5%). This variant was also reported in our recent study [13].

To further understand the genetic structure around this variant and access the founder event, we performed whole genome array genotyping of 68 patients and compared them with individuals with no LGMD phenotype. In the past, homozygous pathogenic variants have been reported in several nonconsanguineous families. For example, in mucopolysaccharidosis I, 54.6% of probands were from nonconsanguineous family [32], and for MONA, all probands were from nonconsanguineous family [33]. Recently, a large ROH around homozygous mutations in autosomal recessive disorders has been reported in Indian nonconsanguineous families [34]. This suggests that the homozygous pathogenic variants in nonconsanguineous parents have probably originated from a founder ancestor. This could be because of a unique practice in India across several centuries where marriages are done among the same caste, leading to a type of inbreeding. Due to this unique practice of marriage practice, the presence of founder mutation is higher in certain groups and communities in India [35].

Our analysis revealed a ~1 Mb (chr4:51817441-528499552) ROH region encompassing 133 array markers and 6 protein-coding genes—*DCUN1D4*, *LRRC66*, *SGCB*, *SPATA18*, *USP46*, and *ERVMER34-1*. So far, large ROH in a large proportion is not reported in previous LGMD and muscular diseases. Like reported in previous studies, many of these patients (37%) are from nonconsanguineous families. Ancestry analysis using admixture suggests a higher proportion of West Eurasian/European ancestry in cases compared to random controls. Haplotype analysis and frequency in the population databases indicate a probable event of the founder event. Further studies are needed to identify the communities and regions in India and other countries of South Asia where the *SGCB* p.Thr182Pro variant is observed in higher proportions. We also recommend that this variant should be included as part of genetic screening along with other variants that are being screened.

## Figures and Tables

**Table 1:** Frequency of 7 markers with alternative allele as major allele found in common ROH region.

Chrom	Position (GRCh38)	rsid	Ref-base	Alt-base	Case (*N* = 68)	Control (*N* = 128)	*p* value	Population level databases		
								gnomAD v3.1.2	1000G	GA100K
4	51817441	rs1910739	T	C	0.8529	0.5586	<0.0001	0.3659	0.5306	0.5023
4	51818654	rs2874073	A	C	0.8529	0.8398	0.7717	0.8694	0.8970	0.8907
4	51918512	rs6852236	A	G	0.8529	0.5664	<0.0001	0.4132	0.5164	0.5181
4	52102311	rs13122418	G	A	0.8529	0.6250	<0.0001	0.6235	0.5445	0.5941
4	52117235	rs11133358	A	G	0.8529	0.9883	0.4599	0.8038	0.7859	0.9543
4	52195229	rs13353646	C	T	0.8529	0.3945	<0.0001	0.1746	0.1933	0.2177
4	52269957	rs6554360	C	T	0.8382	0.6094	<0.0001	0.4297	0.4860	0.5800

## Data Availability

The results used to support the findings of this study are included within the supplementary. The additional data used to support the findings of this study can be provided on requests to Ravi Gupta (ravig@medgenome.com).

## Supplementary Materials

Supplementary Materials Table S1: list of OMIM terms that captured the patient phenotype(s) and its frequency in proband. Table S2: list of gene and total homozygous and compound heterozygous cases identified in our cohort. Table S3: (a) maternal haplogroup identified in case and control and (b) Y haplogroup identified in case and control. Table S4: ROH identified in the samples overlapping variant of interest. Table S5: list of samples with single or double allele identified after haplotype analysis. Table S6: list of 133 marker haplotypes and its frequency in case and control.
