# Supplementary material for: Large Region of Homozygous (ROH) Identified in Indian Patients with Autosomal Recessive Limb-Girdle Muscular Dystrophy with p.Thr182Pro Variant in SGCB Gene
Source: Hum Mutat. 2023 Mar 28;2023:4362273. doi: 10.1155/2023/4362273 (PMC11918903; doi:10.1155/2023/4362273)
Supplement: Supplementary Materials — Table S1: list of OMIM terms that captured the patient phenotype(s) and its frequency in proband. Table S2: list of gene and total homozygous and compound heterozygous cases identified in our cohort. Table S3: (a) maternal haplogroup identified in case and control and (b) Y haplogroup identified in case and control. Table S4: ROH identified in the samples overlapping variant of interest. Table S5: list of samples with single or double allele identified after haplotype analysis. Table S6: list of 133 marker haplotypes and its frequency in case and control. [file 4362273.f1.pdf]

Table #	Description
**Table S1**	List of OMIM terms that captured the patient phenotype(s) and it's frequency in proband
**Table S2**	List of gene and total homozygous and compound heterozygous cases identified in our cohort
**Table S3**	(a) Maternal Haplogroup identified in case and control, (b) Y Haplogroup identified in case and control
**Table S4**	ROH identified in the samples overlapping variant of interest
**Table S5**	List of samples with single or double allele identified after haplotype analysis
**Table S6**	List of 133 markers haplotype and its frequency in case and control

**Table S1.** List of OMIM terms that captured the patient phenotype(s) and it's frequency in proband			
**OMIM Term**	**OMIM ID**	**# of probands**	**% of proband**
MUSCULAR DYSTROPHY, LIMB-GIRDLE, AUTOSOMAL RECESSIVE 1; LGMDR1	253600	206	19.694
MUSCULAR DYSTROPHY, LIMB-GIRDLE, AUTOSOMAL RECESSIVE 4; LGMDR4	604286	199	19.025
MUSCULAR DYSTROPHY, LIMB-GIRDLE, AUTOSOMAL RECESSIVE 2; LGMDR2	253601	183	17.495
MIYOSHI MUSCULAR DYSTROPHY 1; MMD1	254130	152	14.532
ULLRICH CONGENITAL MUSCULAR DYSTROPHY 1; UCMD1	254090	90	8.6042
MYOPATHY, DISTAL, WITH ANTERIOR TIBIAL ONSET; DMAT	606768	84	8.0306
MUSCULAR DYSTROPHY, LIMB-GIRDLE, AUTOSOMAL RECESSIVE 3; LGMDR3	608099	78	7.457
BETHLEM MYOPATHY 1; BTHLM1	158810	60	5.7361
MUSCULAR DYSTROPHY, LIMB-GIRDLE, AUTOSOMAL RECESSIVE 5; LGMDR5	253700	42	4.0153
MUSCULAR DYSTROPHY-DYSTROGLYCANOPATHY (LIMB-GIRDLE), TYPE C, 5; MDDGC5	607155	26	2.4857
MUSCULAR DYSTROPHY-DYSTROGLYCANOPATHY (CONGENITAL WITH OR WITHOUT IMPAIRED INTELLECTUAL DEVELOPMENT), TYPE B, 5; MDDGB5	606612	22	2.1033
MUSCULAR DYSTROPHY-DYSTROGLYCANOPATHY (CONGENITAL WITH BRAIN AND EYE ANOMALIES), TYPE A, 3; MDDGA3	253280	16	1.5296
MUSCULAR DYSTROPHY, LIMB-GIRDLE, AUTOSOMAL RECESSIVE 7; LGMDR7	601954	14	1.3384
MUSCULAR DYSTROPHY-DYSTROGLYCANOPATHY (CONGENITAL WITH BRAIN AND EYE ANOMALIES), TYPE A, 5; MDDGA5	613153	14	1.3384
MUSCULAR DYSTROPHY-DYSTROGLYCANOPATHY (CONGENITAL WITH IMPAIRED INTELLECTUAL DEVELOPMENT), TYPE B, 3; MDDGB3	613151	13	1.2428
MUSCULAR DYSTROPHY-DYSTROGLYCANOPATHY (LIMB-GIRDLE), TYPE C, 1; MDDGC1	609308	12	1.1472
MYOSCLEROSIS, AUTOSOMAL RECESSIVE	255600	10	0.956
MUSCULAR DYSTROPHY, LIMB-GIRDLE, AUTOSOMAL RECESSIVE 6; LGMDR6	601287	10	0.956
MUSCULAR DYSTROPHY, LIMB-GIRDLE, AUTOSOMAL RECESSIVE 10; LGMDR10	608807	9	0.8604
MUSCULAR DYSTROPHY-DYSTROGLYCANOPATHY (CONGENITAL WITH BRAIN AND EYE ANOMALIES), TYPE A, 1; MDDGA1	236670	8	0.7648
MUSCULAR DYSTROPHY-DYSTROGLYCANOPATHY (LIMB-GIRDLE), TYPE C, 4; MDDGC4	611588	8	0.7648
MUSCULAR DYSTROPHY-DYSTROGLYCANOPATHY (LIMB-GIRDLE), TYPE C, 14; MDDGC14	615352	8	0.7648
MUSCULAR DYSTROPHY, LIMB-GIRDLE, AUTOSOMAL DOMINANT 4; LGMDD4	618129	8	0.7648
MUSCULAR DYSTROPHY-DYSTROGLYCANOPATHY (CONGENITAL WITH BRAIN AND EYE ANOMALIES), TYPE A, 4; MDDGA4	253800	7	0.6692
MUSCULAR DYSTROPHY-DYSTROGLYCANOPATHY (LIMB-GIRDLE), TYPE C, 2; MDDGC2	613158	7	0.6692
MUSCULAR DYSTROPHY-DYSTROGLYCANOPATHY (CONGENITAL WITHOUT IMPAIRED INTELLECTUAL DEVELOPMENT), TYPE B, 4; MDDGB4	613152	6	0.5736
MUSCULAR DYSTROPHY-DYSTROGLYCANOPATHY (CONGENITAL WITH IMPAIRED INTELLECTUAL IMPAIRMENT), TYPE B, 1; MDDGB1	613155	6	0.5736
MUSCULAR DYSTROPHY-DYSTROGLYCANOPATHY (LIMB-GIRDLE), TYPE C, 3; MDDGC3	613157	6	0.5736
MUSCULAR DYSTROPHY-DYSTROGLYCANOPATHY (CONGENITAL WITH BRAIN AND EYE ANOMALIES), TYPE A, 2; MDDGA2	613150	5	0.478
MUSCULAR DYSTROPHY-DYSTROGLYCANOPATHY (CONGENITAL WITH IMPAIRED INTELLECTUAL DEVELOPMENT), TYPE B, 2; MDDGB2	613156	5	0.478
MUSCULAR DYSTROPHY-DYSTROGLYCANOPATHY (CONGENITAL WITH IMPAIRED INTELLECTUAL DEVELOPMENT), TYPE B, 14; MDDGB14	615351	5	0.478
MUSCULAR DYSTROPHY-DYSTROGLYCANOPATHY (LIMB-GIRDLE), TYPE C, 7; MDDGC7	616052	5	0.478
EPIDERMOLYSIS BULLOSA SIMPLEX 5D, GENERALIZED INTERMEDIATE, AUTOSOMAL RECESSIVE; EBS5D	616487	5	0.478
MUSCULAR DYSTROPHY, LIMB-GIRDLE, AUTOSOMAL RECESSIVE 23; LGMDR23	618138	5	0.478
EPIDERMOLYSIS BULLOSA SIMPLEX 5B, WITH MUSCULAR DYSTROPHY; EBS5B	226670	4	0.3824
EPIDERMOLYSIS BULLOSA SIMPLEX 5C, WITH PYLORIC ATRESIA; EBS5C	612138	4	0.3824
MUSCULAR DYSTROPHY-DYSTROGLYCANOPATHY (CONGENITAL WITH BRAIN AND EYE ANOMALIES), TYPE A, 14; MDDGA14	615350	4	0.3824
MUSCULAR DYSTROPHY, LIMB-GIRDLE, AUTOSOMAL RECESSIVE 8; LGMDR8	254110	3	0.2868
MUSCULAR DYSTROPHY-DYSTROGLYCANOPATHY (CONGENITAL WITH BRAIN AND EYE ANOMALIES), TYPE A, 7; MDDGA7	614643	3	0.2868
MUSCULAR DYSTROPHY, LIMB-GIRDLE, AUTOSOMAL RECESSIVE 12; LGMDR12	611307	2	0.1912
SALIH MYOPATHY; SALMY	611705	2	0.1912
EMERY-DREIFUSS MUSCULAR DYSTROPHY 3, AUTOSOMAL RECESSIVE; EDMD3	616516	2	0.1912
EMERY-DREIFUSS MUSCULAR DYSTROPHY 2, AUTOSOMAL DOMINANT; EDMD2	181350	1	0.0956
MYOPATHY, MYOFIBRILLAR, 1; MFM1	601419	1	0.0956
CARDIOMYOPATHY, DILATED, 1L; CMD1L	606685	1	0.0956
MUSCULAR DYSTROPHY, CONGENITAL, LMNA-RELATED	613205	1	0.0956
MIYOSHI MUSCULAR DYSTROPHY 3; MMD3	613319	1	0.0956
MUSCULAR DYSTROPHY, LIMB-GIRDLE, AUTOSOMAL RECESSIVE 17; LGMDR17	613723	1	0.0956
MYOPATHY, MYOFIBRILLAR, 1; MFM1	615325	1	0.0956
MUSCULAR DYSTROPHY, LIMB-GIRDLE, AUTOSOMAL RECESSIVE 18; LGMDR18	615356	1	0.0956

Table S2. List of gene and total homozygous and compound heterozygous cases identified in our cohort

Gene	# of homozygous	# of compound het
CAPN3	179	52
SGCB	206	11
DYSF	184	32
SGCA	89	2
SGCG	48	2
COL6A2	39	6
COL6A1	22	4
FKRP	22	3
COL6A3	20	1
POMGNT1	17	0
TCAP	15	0
POMT1	7	7
TTN	11	1
SGCD	12	0
GMPPB	7	4
FKTN	9	1
POMT2	6	2
ISPD	6	0
PLEC	5	1
LAMA2	2	3
TRIM32	4	0
LMNA	2	1
ANO5	1	2
DES	1	0
TRAPPC11	1	0

Supp. Table S3a. Maternal Haplogroup identified in case and control

Haplogroup	# of Controls	# of Cases	% Control	% Cases
H1a3	0	2	0	3.03030303
H1aj	0	1	0	1.515151515
H1ak	1	0	0.819672	0
H1p	1	0	0.819672	0
H44	0	1	0	1.515151515
H46	1	0	0.819672	0
I	1	0	0.819672	0
I1	0	1	0	1.515151515
J1b1	0	2	0	3.03030303
M	9	8	7.377049	12.12121212
M18a	8	1	6.557377	1.515151515
M19'53	0	2	0	3.03030303
M2a'b	12	3	9.836066	4.545454545
M2b1	1	1	0.819672	1.515151515
M3	5	3	4.098361	4.545454545
M30	0	2	0	3.03030303
M35	6	2	4.918033	3.03030303
M35b	2	0	1.639344	0
M36	5	2	4.098361	3.03030303
M36a	0	1	0	1.515151515
M4	1	0	0.819672	0
M40a	1	0	0.819672	0
M40a*	1	1	0.819672	1.515151515
M42b	1	0	0.819672	0
M44	0	1	0	1.515151515
M5	16	5	13.11475	7.575757576
M57	0	2	0	3.03030303
M5a5	1	0	0.819672	0
M6	2	0	1.639344	0
M6a	1	0	0.819672	0
M6a1	1	2	0.819672	3.03030303
M6a1a	12	2	9.836066	3.03030303
M81*	2	1	1.639344	1.515151515
M9	1	0	0.819672	0
N1'5	1	2	0.819672	3.03030303
N1a1	2	0	1.639344	0
P2'10	1	0	0.819672	0
R	1	0	0.819672	0
R30	1	1	0.819672	1.515151515
R30a1*	2	0	1.639344	0
R30b2a	1	0	0.819672	0
R5	2	0	1.639344	0
R5a2a	1	0	0.819672	0
R6+16129	0	1	0	1.515151515
R6a2	1	0	0.819672	0
R7a	0	1	0	1.515151515
R8	1	1	0.819672	1.515151515
T1	0	1	0	1.515151515
U1a1	0	1	0	1.515151515
U1a1c1	0	1	0	1.515151515
U2	4	6	3.278689	9.090909091
U2'3'4'7'8'9	1	0	0.819672	0
U2a2	1	0	0.819672	0
U2c'd	8	2	6.557377	3.03030303
U4b1b	1	0	0.819672	0
U7	0	1	0	1.515151515
U7a3b	1	0	0.819672	0
W1c	0	1	0	1.515151515
W3a1	0	1	0	1.515151515
**Total**	122	66		

Supp. Table S3b. Y Haplogroup identified in case and control

Y Haplogroup	# of Control	# of Cases	% Control	% Cases
H1a1	10	14	20.40816	28
R1a1	4	10	8.163265	20
R2a2	4	8	8.163265	16
J2a1	2	5	4.081633	10
L1a1	7	4	14.28571	8
H3b'	0	4	0	8
H1a'	2	2	4.081633	4
G1a2	1	1	2.040816	2
J2a2	0	1	0	2
J2b2	6	0	12.2449	0
H1a2	3	0	6.122449	0
G2a2	3	0	6.122449	0
C1b'	1	0	2.040816	0
H3',	1	1	2.040816	2
Q2b'	1	0	2.040816	0
L1a'	3	0	6.122449	0
Q1a2	1	0	2.040816	0
**Total**	**49**	**50**		

Supp. Table S4. ROH identified in the samples overlapping variant of interest

SampleID	Chromosome	Start	End	Length (Kb)	# of SNP	Group
311254	4	51817441	52745501	928.061	119	Case
309614	4	51817441	52846137	1028.697	131	Case
297188	4	51817441	52873505	1056.065	137	Case
143073	4	51817441	52926311	1108.871	143	Case
245074	4	51817441	53003498	1186.058	151	Case
287869	4	51817441	53003498	1186.058	151	Case
291799	4	51817441	53006507	1189.067	153	Case
351193	4	51817441	53006507	1189.067	153	Case
146193	4	51817441	53793388	1975.948	272	Case
324200	4	51817441	53817855	2000.415	277	Case
183492	4	51817441	54101542	2284.102	321	Case
124718	4	51817441	54312942	2495.502	359	Case
254967	4	51817441	54583449	2766.009	418	Case
111929	4	51817441	54744682	2927.242	464	Case
151727	4	51817441	54758192	2940.752	468	Case
224758	4	51817441	54842249	3024.809	487	Case
145907	4	51817441	54950315	3132.875	513	Case
250567	4	51817441	54959211	3141.771	515	Case
339883	4	51817441	55020392	3202.952	528	Case
247811	4	51817441	55080504	3263.064	537	Case
80736	4	51817441	55115364	3297.924	562	Case
107450	4	51817441	55119648	3302.208	564	Case
171330	4	51817441	55768468	3951.028	677	Case
111937	4	51817441	56116075	4298.635	754	Case
126074	4	51817441	56188396	4370.956	775	Case
130683	4	51817441	56240029	4422.589	787	Case
327171	4	51817441	56296453	4479.013	806	Case
292081	4	51817441	56301114	4483.674	808	Case
289340	4	51817441	56641270	4823.83	880	Case
239050	4	51817441	56670154	4852.714	889	Case
97194	4	51817441	56681900	4864.46	890	Case
219735	4	51817441	56821191	5003.751	927	Case
319009	4	51817441	56841303	5023.863	934	Case
97177	4	51817441	57163034	5345.594	1017	Case
258771	4	51817441	57273907	5456.467	1048	Case
148316	4	51817441	57397700	5580.26	1078	Case
153498	4	51817441	60341335	8523.895	1555	Case
155188	4	51817441	60342766	8525.326	1556	Case
300730	4	51817441	60606957	8789.517	1604	Case
219039	4	51817441	61109815	9292.375	1716	Case
400018	4	51817441	61186749	9369.309	1730	Case
300769	4	51817441	63648599	11831.159	2154	Case
51106	4	51817441	63784448	11967.008	2172	Case
577094	4	51817441	65300731	13483.291	2387	Case
579632	4	51817441	65734601	13917.161	2490	Case
111910	4	51817441	65734601	13917.161	2490	Case
111947	4	51817441	66880571	15063.131	2655	Case
287894	4	51817441	68210494	16393.054	2877	Case
268715	4	51817441	68390234	16572.794	2905	Case
132245	4	51817441	68500812	16683.372	2928	Case
132252	4	51817441	68535454	16718.014	2929	Case
145362	4	51817441	68535454	16718.014	2929	Case
145776	4	51817441	68535454	16718.014	2929	Case
145777	4	51817441	68535454	16718.014	2929	Case
151752	4	51817441	68535454	16718.014	2929	Case
179870	4	51817441	68535454	16718.014	2929	Case
231641	4	51817441	68535454	16718.014	2929	Case
267148	4	51817441	68535454	16718.014	2929	Case
268986	4	51817441	68535454	16718.014	2929	Case
287863	4	51817441	68535454	16718.014	2929	Case
319643	4	51817441	68535454	16718.014	2929	Case
324539	4	51817441	68535454	16718.014	2929	Case
357199	4	51817441	68535454	16718.014	2929	Case
358781	4	51817441	68535454	16718.014	2929	Case
77113	4	51817441	68535454	16718.014	2929	Case
107448	4	51817441	68535454	16718.014	2929	Case
107475	4	51817441	68535454	16718.014	2929	Case
97168	4	51817441	68535454	16718.014	2929	Case
589355	4	51865294	52197055	331.762	55	Control
589452	4	51998310	52351306	352.997	51	Control
588737	4	51817441	52186066	368.626	55	Control
589427	4	51865294	52234673	369.38	56	Control
588778	4	51817441	52234673	417.233	60	Control
589524	4	51817441	52255543	438.103	61	Control
588860	4	51865294	52351306	486.013	67	Control
146190	4	51865294	52351306	486.013	67	Control
588730	4	51817441	52351306	533.866	71	Control
589171	4	51817441	52351306	533.866	71	Control
148318	4	51817441	52351306	533.866	71	Control
589583	4	51817441	52373437	555.997	75	Control
589224	4	51853931	52422110	568.18	78	Control
588727	4	51817441	52403845	586.405	76	Control
588797	4	51817441	52407124	589.684	77	Control
589257	4	51817441	52416927	599.487	80	Control
589350	4	51817441	52422110	604.67	81	Control
589379	4	51817441	52533450	716.01	94	Control
588766	4	51817441	52549581	732.141	98	Control
588964	4	51945249	52923518	978.27	127	Control
588559	4	51865294	52849552	984.259	129	Control
589491	4	51853931	52873505	1019.575	134	Control
153499	4	51817441	52846137	1028.697	131	Control
588714	4	51817441	52869152	1051.712	135	Control
589156	4	51817441	52998996	1181.556	150	Control
589358	4	51817441	53006507	1189.067	153	Control
588807	4	51817441	53051209	1233.769	160	Control
589573	4	51817441	54560744	2743.304	412	Control
588821	4	51817441	60103404	8285.964	1525	Control
588788	4	51817441	68232288	16414.848	2883	Control

Supp. Table S5. List of samples with single or double allele identified after haplotype analysis

Sample IDs	Single/Double Allele	Phenotype
588580	S	Control
588586	S	Control
588651	S	Control
588668	S	Control
588680	S	Control
588714	S	Control
**588727 (0.113)**	S	Control
**588731 (0.037)**	S	Control
588797	S	Control
588807	S	Control
588816	S	Control
588860	S	Control
588971	S	Control
589132	S	Control
589148	S	Control
**589203 (0.341)**	S	Control
**589355 (0.061)**	S	Control
589417	S	Control
589427	S	Control
**589452 (0.393)**	S	Control
**589540 (0.065)**	S	Control
145778	S	Control
146190	S	Control
148317	S	Control
148318	S	Control
151728	S	Control
151753	S	Control
153499	S	Control
158799	S	Control
158800	S	Control
145777	D	Case
124718	D	Case
126074	D	Case
130683	D	Case
132245	D	Case
132252	D	Case
143073	D	Case
145362	D	Case
145776	D	Case
145907	D	Case
146193	D	Case
148316	D	Case
151727	D	Case
151752	D	Case
153498	D	Case
155188	D	Case
171330	D	Case
183492	D	Case
219039	D	Case
219735	D	Case
224758	D	Case
231641	D	Case
239050	D	Case
245074	D	Case
247811	D	Case
250567	D	Case
254967	D	Case
258771	D	Case
267148	D	Case
268715	D	Case
268986	D	Case
287863	D	Case
287869	D	Case
287894	D	Case
289340	D	Case
291799	D	Case
292081	D	Case
300730	D	Case
300769	D	Case
319009	D	Case
319643	D	Case
324200	D	Case
324539	D	Case
327171	D	Case
339883	D	Case
351193	D	Case
358781	D	Case
400018	D	Case
51106	D	Case
577094	D	Case
579632	D	Case
77113	D	Case
80736	D	Case
107448	D	Case
107450	D	Case
107475	D	Case
111910	D	Case
111929	D	Case
111947	D	Case
97168	D	Case
97177	D	Case
97194	D	Case
309614	S	Case
311254	S	Case

Supp. Table S6. List of 133 markers haplotype and its frequency in case and control

Chrom	Position (GRCh38)	rsid	RefBase	AltBase	# of cases	# of controls	Hom-Case	Het-Case	NoCall-Case	Hom-Control	Het-Control	NoCall-Control	Hom-Case %	Hom-Control %	Alt Allele Fraction in Case	Alt Allele Fraction in Contr
4	51817441	rs1910739	T	C	68	128	58	0	0	39	65	0	85.294	30.47	0.85294	0.559
4	51818654	rs2874073	A	C	68	128	58	0	0	91	33	0	85.294	71.09	0.85294	0.84
4	51823588	GSA-rs1855	A	G	68	128	0	0	0	0	0	1	0	0	0	0
4	51853931	GSA-rs7555	G	A	68	128	0	0	0	0	2	0	0	0	0	0.008
4	51865294	GSA-rs1849	C	T	68	128	0	0	0	0	0	0	0	0	0	0
4	51871985	GSA-rs1894	G	A	68	128	0	0	0	0	0	0	0	0	0	0
4	51878172	GSA-rs7469	A	G	68	128	0	0	0	0	0	0	0	0	0	0
4	51883723	GSA-rs3414	A	C	68	128	0	0	0	0	0	0	0	0	0	0
4	51890254	GSA-rs7159	T	G	68	128	0	0	0	0	0	0	0	0	0	0
4	51891638	GSA-rs1705	A	G	68	128	0	0	0	0	3	0	0	0	0	0.012
4	51912644	GSA-rs5612	G	A	68	128	0	0	0	0	0	0	0	0	0	0
4	51914022	rs17082071	T	G	68	128	0	0	0	0	2	0	0	0	0	0.008
4	51918512	rs6852236	A	G	68	128	58	0	0	40	65	0	85.294	31.25	0.85294	0.566
4	51921806	GSA-rs1747	C	T	68	128	0	0	0	0	5	0	0	0	0	0.02
4	51922572	GSA-rs7992	T	C	68	128	0	0	0	0	0	0	0	0	0	0
4	51945249	GSA-rs7933	G	T	68	128	0	0	0	0	0	0	0	0	0	0
4	51963334	GSA-rs7981	C	T	68	128	0	0	0	0	0	0	0	0	0	0
4	51980612	GSA-rs1519	A	G	68	128	0	0	0	2	20	0	0	1.563	0	0.094
4	51981814	GSA-rs5780	C	T	68	128	0	0	0	2	12	0	0	1.563	0	0.063
4	51992222	GSA-rs7621	T	C	68	128	0	0	0	0	1	0	0	0	0	0.004
4	51998310	GSA-rs1761	C	T	68	128	0	0	0	0	2	0	0	0	0	0.008
4	52003339	GSA-rs3737	A	C	68	128	0	0	0	0	0	0	0	0	0	0
4	52005059	GSA-rs1146	G	A	68	128	0	0	0	1	3	0	0	0.781	0	0.02
4	52022852	GSA-rs7740	T	C	68	128	0	0	0	0	0	0	0	0	0	0
4	52023971	rs150395645	C	T	68	128	0	0	0	0	0	0	0	0	0	0
4	52024115	rs200761715	G	A	68	128	0	0	0	1	4	1	0	0.781	0	0.023
4	52028799	rs104893868	A	C	68	128	0	0	0	0	0	0	0	0	0	0
4	52028899	rs28936383	G	C	68	128	0	0	0	0	0	0	0	0	0	0
4	52029766	rs150518260	G	A	68	128	0	0	0	0	0	0	0	0	0	0
4	52029808	rs104893871	A	T	68	128	0	0	0	0	0	0	0	0	0	0
4	52029835	rs104893869	C	G	68	128	0	0	0	0	0	0	0	0	0	0
4	52033523	rs144743676	G	A	68	128	0	0	0	0	0	0	0	0	0	0
4	52036699	GSA-rs1163	G	T	68	128	0	0	0	0	5	0	0	0	0	0.02
4	52038259	rs398123262	T	C	68	128	0	0	0	0	0	0	0	0	0	0
4	52046759	GSA-rs7678	G	A	68	128	0	0	0	0	1	0	0	0	0	0.004
4	52048068	rs12502690	C	A	68	128	0	0	0	7	41	0	0	5.469	0	0.215
4	52054457	GSA-rs7324	G	A	68	128	0	0	0	0	0	0	0	0	0	0
4	52067977	GSA-rs1425	A	G	68	128	0	0	0	0	0	0	0	0	0	0
4	52071006	GSA-rs7973	T	C	68	128	0	0	0	0	0	0	0	0	0	0
4	52072079	GSA-rs7925	C	T	68	128	0	0	0	0	0	0	0	0	0	0
4	52076895	GSA-rs1416	A	G	68	128	0	0	0	0	0	0	0	0	0	0
4	52097928	GSA-rs1765	A	G	68	128	0	0	0	3	33	0	0	2.344	0	0.152
4	52102311	GSA-rs1312	G	A	68	128	58	0	0	53	54	0	85.294	41.41	0.85294	0.625
4	52103566	rs11722603	A	G	68	128	0	0	0	6	48	0	0	4.688	0	0.234
4	52106536	rs7692196	A	G	68	128	0	0	0	10	48	0	0	7.813	0	0.266
4	52113828	GSA-rs1379	C	A	68	128	0	0	0	0	0	0	0	0	0	0
4	52114270	rs77257596	G	A	68	128	0	0	0	0	2	0	0	0	0	0.008
4	52117235	exm2265838	A	G	68	128	58	0	0	125	3	0	85.294	97.66	0.85294	0.988
4	52128086	GSA-rs8032	A	G	68	128	0	0	0	0	3	0	0	0	0	0.012
4	52135100	GSA-rs1858	T	C	68	128	0	0	0	0	0	0	0	0	0	0
4	52163905	GSA-rs7810	G	A	68	128	0	0	0	0	0	0	0	0	0	0
4	52172391	rs10517246	T	C	68	128	0	0	0	18	58	0	0	14.06	0	0.367
4	52173895	GSA-rs1445	C	T	68	128	0	0	0	0	1	0	0	0	0	0.004
4	52181123	GSA-rs7695	C	T	68	128	0	0	0	0	1	0	0	0	0	0.004
4	52186066	GSA-rs1140	G	A	68	128	0	0	0	0	0	0	0	0	0	0
4	52187465	GSA-rs1149	G	A	68	128	0	0	0	0	7	0	0	0	0	0.027
4	52191793	GSA-rs1927	T	C	68	128	0	0	0	0	0	0	0	0	0	0
4	52195229	rs13353646	C	T	68	128	58	0	0	17	67	0	85.294	13.28	0.85294	0.395
4	52197055	GSA-rs1747	A	G	68	128	0	0	0	0	1	0	0	0	0	0.004
4	52234673	GSA-rs1178	G	T	68	128	0	0	0	0	0	0	0	0	0	0
4	52255543	GSA-rs7866	T	G	68	128	0	0	0	0	0	0	0	0	0	0
4	52256766	rs11731574	T	C	68	128	1	0	0	6	41	0	1.4706	4.688	0.01471	0.207
4	52259381	rs1351556	A	G	68	128	1	2	0	34	69	0	1.4706	26.56	0.02941	0.535
4	52269957	GSA-rs6554	C	T	68	128	57	0	0	45	66	0	83.824	35.16	0.83824	0.609
4	52271173	GSA-rs1123	T	G	68	128	0	0	0	0	3	0	0	0	0	0.012
4	52295869	GSA-rs1151	G	A	68	128	0	0	0	0	3	0	0	0	0	0.012
4	52305367	GSA-rs1149	A	G	68	128	0	0	0	0	0	0	0	0	0	0
4	52312344	GSA-rs7499	A	G	68	128	0	0	0	0	0	0	0	0	0	0
4	52335169	GSA-rs1166	G	A	68	128	0	0	0	0	0	0	0	0	0	0
4	52344118	GSA-rs1920	G	A	68	128	0	0	0	0	0	0	0	0	0	0
4	52351306	GSA-rs7159	C	T	68	128	0	0	0	0	0	0	0	0	0	0
4	52356308	GSA-rs1816	A	G	68	128	0	0	0	0	0	0	0	0	0	0
4	52358522	GSA-rs7801	C	T	68	128	0	0	0	0	0	0	0	0	0	0
4	52367953	GSA-rs1250	G	T	68	128	1	0	0	19	61	0	1.4706	14.84	0.01471	0.387
4	52373437	rs1459814	A	G	68	128	1	0	0	20	60	0	1.4706	15.63	0.01471	0.391
4	52403845	GSA-rs7692	C	T	68	128	0	0	0	6	51	0	0	4.688	0	0.246
4	52407124	GSA-rs1865	C	A	68	128	0	0	0	0	0	0	0	0	0	0
4	52407216	GSA-rs1757	C	A	68	128	0	0	0	0	2	0	0	0	0	0.008
4	52407640	rs10027172	C	A	68	128	1	0	0	1	18	0	1.4706	0.781	0.01471	0.078
4	52416927	GSA-rs6834	T	C	68	128	2	0	0	22	60	0	2.9412	17.19	0.02941	0.406
4	52422110	rs11133542	G	A	68	128	1	0	0	12	60	0	1.4706	9.375	0.01471	0.328
4	52424092	GSA-rs7479	A	G	68	128	0	0	0	0	1	0	0	0	0	0.004
4	52431769	GSA-rs2411	G	T	68	128	0	0	0	5	33	0	0	3.906	0	0.168
4	52440695	GSA-rs1459	C	A	68	128	2	0	0	30	59	0	2.9412	23.44	0.02941	0.465
4	52458856	rs11732165	A	G	68	128	1	0	0	15	61	1	1.4706	11.72	0.01471	0.355
4	52470129	GSA-rs1149	G	A	68	128	0	0	0	0	1	0	0	0	0	0.004
4	52483710	GSA-rs1142	G	T	68	128	0	0	0	0	2	0	0	0	0	0.008
4	52505150	GSA-rs1511	A	G	68	128	0	0	0	0	0	0	0	0	0	0
4	52505960	GSA-rs1761	C	T	68	128	0	0	0	0	0	0	0	0	0	0
4	52506537	GSA-rs7999	C	T	68	128	0	0	0	0	0	0	0	0	0	0
4	52508948	GSA-rs1163	G	A	68	128	0	0	0	0	3	0	0	0	0	0.012
4	52516963	rs346901	A	G	68	128	2	0	0	29	59	0	2.9412	22.66	0.02941	0.457
4	52521018	GSA-rs1150	G	T	68	128	0	0	0	0	0	0	0	0	0	0
4	52533450	rs1347763	T	C	68	128	1	0	0	4	20	0	1.4706	3.125	0.01471	0.109
4	52537349	GSA-rs1151	C	T	68	128	0	0	0	0	3	0	0	0	0	0.012
4	52538131	GSA-rs1173	A	G	68	128	0	0	0	0	0	0	0	0	0	0
4	52546136	GSA-rs1264	A	G	68	128	0	0	0	1	22	0	0	0.781	0	0.094
4	52549581	GSA-rs7854	A	G	68	128	0	0	0	0	0	0	0	0	0	0
4	52555506	GSA-rs7324	A	G	68	128	0	0	0	0	0	0	0	0	0	0
4	52565654	GSA-rs7744	G	A	68	128	0	0	0	0	0	0	0	0	0	0
4	52568445	rs346011	A	G	68	128	0	1	0	3	29	0	0	2.344	0.00735	0.137
4	52572299	GSA-rs1385	T	C	68	128	0	0	0	0	1	0	0	0	0	0.004
4	52600437	GSA-rs1423	C	T	68	128	0	0	0	0	0	0	0	0	0	0
4	52617584	GSA-rs7956	C	T	68	128	0	0	0	0	0	1	0	0	0	0
4	52619284	GSA-rs1144	A	G	68	128	0	0	0	0	0	0	0	0	0	0
4	52625174	GSA-rs7324	C	T	68	128	0	0	0	0	1	0	0	0	0	0.004
4	52642185	GSA-rs1143	T	C	68	128	0	0	0	0	0	0	0	0	0	0
4	52663432	GSA-rs1475	T	C	68	128	0	0	0	0	0	0	0	0	0	0
4	52663941	GSA-rs1153	T	C	68	128	0	0	0	0	0	0	0	0	0	0
4	52666714	GSA-rs1061	T	C	68	128	0	0	0	0	0	0	0	0	0	0
4	52669620	GSA-rs1449	G	A	68	128	0	0	0	0	0	0	0	0	0	0
4	52675503	rs7678171	G	A	68	128	0	0	0	0	17	0	0	0	0	0.066
4	52682083	GSA-rs7571	A	G	68	128	0	0	0	0	1	1	0	0	0	0.004
4	52685260	GSA-rs7737	T	C	68	128	0	0	0	0	5	0	0	0	0	0.02
4	52711842	GSA-rs1002	G	A	68	128	0	0	0	2	18	0	0	1.563	0	0.086
4	52712142	GSA-rs7324	G	A	68	128	0	0	0	0	1	1	0	0	0	0.004
4	52712204	rs3811779	T	G	68	128	0	0	0	0	3	0	0	0	0	0.012
4	52743130	GSA-rs1472	C	A	68	128	0	0	0	0	0	0	0	0	0	0
4	52745501	rs3733504	T	C	68	128	0	0	0	2	17	0	0	1.563	0	0.082
4	52750459	GSA-rs7621	A	G	68	128	0	0	0	0	2	0	0	0	0	0.008
4	52753200	GSA-rs4865	C	T	68	128	0	0	0	2	15	0	0	1.563	0	0.074
4	52755569	GSA-rs1310	G	A	68	128	0	1	0	0	2	0	0	0	0.00735	0.008
4	52757282	GSA-rs9999	G	A	68	128	0	0	0	2	15	0	0	1.563	0	0.074
4	52763020	rs4864672	A	G	68	128	1	0	0	10	47	0	1.4706	7.813	0.01471	0.262
4	52777312	rs2411265	A	G	68	128	1	0	0	9	47	0	1.4706	7.031	0.01471	0.254
4	52800522	GSA-rs7638	G	A	68	128	0	1	0	0	0	0	0	0	0.00735	0
4	52810167	GSA-rs7815	A	G	68	128	0	0	0	1	10	0	0	0.781	0	0.047
4	52834306	GSA-rs1405	A	G	68	128	0	0	0	0	0	0	0	0	0	0
4	52839628	GSA-rs1165	G	T	68	128	0	0	0	0	0	0	0	0	0	0
4	52845904	rs9993034	C	T	68	128	0	0	0	0	6	0	0	0	0	0.023
4	52846137	rs7662817	G	A	68	128	1	0	0	5	34	0	1.4706	3.906	0.01471	0.172
4	52846805	GSA-rs7325	A	C	68	128	0	0	0	0	0	0	0	0	0	0
4	52849552	GSA-4:5371	A	G	68	128	0	0	0	0	0	0	0	0	0	0

4	52197055	GSA-rs1747	A	G	68	128	0	0	0	0	1	0	0	0	0	0.004
4	52234673	GSA-rs1178	G	T	68	128	0	0	0	0	0	0	0	0	0	0
4	52255543	GSA-rs7866	T	G	68	128	0	0	0	0	0	0	0	0	0	0
4	52256766	rs11731574	T	C	68	128	1	0	0	6	41	0	1.4706	4.688	0.01471	0.207
4	52259381	rs1351556	A	G	68	128	1	2	0	34	69	0	1.4706	26.56	0.02941	0.535
4	52269957	GSA-rs6554	C	T	68	128	57	0	0	45	66	0	83.824	35.16	0.83824	0.609
4	52271173	GSA-rs1123	T	G	68	128	0	0	0	0	3	0	0	0	0	0.012
4	52295869	GSA-rs1151	G	A	68	128	0	0	0	0	3	0	0	0	0	0.012
4	52305367	GSA-rs1149	A	G	68	128	0	0	0	0	0	0	0	0	0	0
4	52312344	GSA-rs7499	A	G	68	128	0	0	0	0	0	0	0	0	0	0
4	52335169	GSA-rs1166	G	A	68	128	0	0	0	0	0	0	0	0	0	0
4	52344118	GSA-rs1920	G	A	68	128	0	0	0	0	0	0	0	0	0	0
4	52351306	GSA-rs7159	C	T	68	128	0	0	0	0	0	0	0	0	0	0
4	52356308	GSA-rs1816	A	G	68	128	0	0	0	0	0	0	0	0	0	0
4	52358522	GSA-rs7801	C	T	68	128	0	0	0	0	0	0	0	0	0	0
4	52367953	GSA-rs1250	G	T	68	128	1	0	0	19	61	0	1.4706	14.84	0.01471	0.387
4	52373437	rs1459814	A	G	68	128	1	0	0	20	60	0	1.4706	15.63	0.01471	0.391
4	52403845	GSA-rs7692	C	T	68	128	0	0	0	6	51	0	0	4.688	0	0.246
4	52407124	GSA-rs1865	C	A	68	128	0	0	0	0	0	0	0	0	0	0
4	52407216	GSA-rs1757	C	A	68	128	0	0	0	0	2	0	0	0	0	0.008
4	52407640	rs10027172	C	A	68	128	1	0	0	1	18	0	1.4706	0.781	0.01471	0.078
4	52416927	GSA-rs6834	T	C	68	128	2	0	0	22	60	0	2.9412	17.19	0.02941	0.406
4	52422110	rs11133542	G	A	68	128	1	0	0	12	60	0	1.4706	9.375	0.01471	0.328
4	52424092	GSA-rs7479	A	G	68	128	0	0	0	0	1	0	0	0	0	0.004
4	52431769	GSA-rs2411	G	T	68	128	0	0	0	5	33	0	0	3.906	0	0.168
4	52440695	GSA-rs1459	C	A	68	128	2	0	0	30	59	0	2.9412	23.44	0.02941	0.465
4	52458856	rs11732165	A	G	68	128	1	0	0	15	61	1	1.4706	11.72	0.01471	0.355
4	52470129	GSA-rs1149	G	A	68	128	0	0	0	0	1	0	0	0	0	0.004
4	52483710	GSA-rs1142	G	T	68	128	0	0	0	0	2	0	0	0	0	0.008
4	52505150	GSA-rs1511	A	G	68	128	0	0	0	0	0	0	0	0	0	0
4	52505960	GSA-rs1761	C	T	68	128	0	0	0	0	0	0	0	0	0	0
4	52506537	GSA-rs7999	C	T	68	128	0	0	0	0	0	0	0	0	0	0
4	52508948	GSA-rs1163	G	A	68	128	0	0	0	0	3	0	0	0	0	0.012
4	52516963	rs346901	A	G	68	128	2	0	0	29	59	0	2.9412	22.66	0.02941	0.457
4	52521018	GSA-rs1150	G	T	68	128	0	0	0	0	0	0	0	0	0	0
4	52533450	rs1347763	T	C	68	128	1	0	0	4	20	0	1.4706	3.125	0.01471	0.109
4	52537349	GSA-rs1151	C	T	68	128	0	0	0	0	3	0	0	0	0	0.012
4	52538131	GSA-rs1173	A	G	68	128	0	0	0	0	0	0	0	0	0	0
4	52546136	GSA-rs1264	A	G	68	128	0	0	0	1	22	0	0	0.781	0	0.094
4	52549581	GSA-rs7854	A	G	68	128	0	0	0	0	0	0	0	0	0	0
4	52555506	GSA-rs7324	A	G	68	128	0	0	0	0	0	0	0	0	0	0
4	52565654	GSA-rs7744	G	A	68	128	0	0	0	0	0	0	0	0	0	0
4	52568445	rs346011	A	G	68	128	0	1	0	3	29	0	0	2.344	0.00735	0.137
4	52572299	GSA-rs1385	T	C	68	128	0	0	0	0	1	0	0	0	0	0.004
4	52600437	GSA-rs1423	C	T	68	128	0	0	0	0	0	0	0	0	0	0
4	52617584	GSA-rs7956	C	T	68	128	0	0	0	0	0	1	0	0	0	0
4	52619284	GSA-rs1144	A	G	68	128	0	0	0	0	0	0	0	0	0	0
4	52625174	GSA-rs7324	C	T	68	128	0	0	0	0	1	0	0	0	0	0.004
4	52642185	GSA-rs1143	T	C	68	128	0	0	0	0	0	0	0	0	0	0
4	52663432	GSA-rs1475	T	C	68	128	0	0	0	0	0	0	0	0	0	0
4	52663941	GSA-rs1153	T	C	68	128	0	0	0	0	0	0	0	0	0	0
4	52666714	GSA-rs1061	T	C	68	128	0	0	0	0	0	0	0	0	0	0
4	52669620	GSA-rs1449	G	A	68	128	0	0	0	0	0	0	0	0	0	0
4	52675503	rs7678171	G	A	68	128	0	0	0	0	17	0	0	0	0	0.066
4	52682083	GSA-rs7571	A	G	68	128	0	0	0	0	1	1	0	0	0	0.004
4	52685260	GSA-rs7737	T	C	68	128	0	0	0	0	5	0	0	0	0	0.02
4	52711842	GSA-rs1002	G	A	68	128	0	0	0	2	18	0	0	1.563	0	0.086
4	52712142	GSA-rs7324	G	A	68	128	0	0	0	0	1	1	0	0	0	0.004
4	52712204	rs3811779	T	G	68	128	0	0	0	0	3	0	0	0	0	0.012
4	52743130	GSA-rs1472	C	A	68	128	0	0	0	0	0	0	0	0	0	0
4	52745501	rs3733504	T	C	68	128	0	0	0	2	17	0	0	1.563	0	0.082
4	52750459	GSA-rs7621	A	G	68	128	0	0	0	0	2	0	0	0	0	0.008
4	52753200	GSA-rs4865	C	T	68	128	0	0	0	2	15	0	0	1.563	0	0.074
4	52755569	GSA-rs1310	G	A	68	128	0	1	0	0	2	0	0	0	0.00735	0.008
4	52757282	GSA-rs9999	G	A	68	128	0	0	0	2	15	0	0	1.563	0	0.074
4	52763020	rs4864672	A	G	68	128	1	0	0	10	47	0	1.4706	7.813	0.01471	0.262
4	52777312	rs2411265	A	G	68	128	1	0	0	9	47	0	1.4706	7.031	0.01471	0.254
4	52800522	GSA-rs7638	G	A	68	128	0	1	0	0	0	0	0	0	0.00735	0
4	52810167	GSA-rs7815	A	G	68	128	0	0	0	1	10	0	0	0.781	0	0.047
4	52834306	GSA-rs1405	A	G	68	128	0	0	0	0	0	0	0	0	0	0
4	52839628	GSA-rs1165	G	T	68	128	0	0	0	0	0	0	0	0	0	0
4	52845904	rs9993034	C	T	68	128	0	0	0	0	6	0	0	0	0	0.023
4	52846137	rs7662817	G	A	68	128	1	0	0	5	34	0	1.4706	3.906	0.01471	0.172
4	52846805	GSA-rs7325	A	C	68	128	0	0	0	0	0	0	0	0	0	0
4	52849552	GSA-4:5371	A	G	68	128	0	0	0	0	0	0	0	0	0	0
